# Early Stage Glycosylation Biomarkers in Alzheimer’s Disease

**DOI:** 10.3390/medicines6030092

**Published:** 2019-09-03

**Authors:** Patricia Regan, Paula L. McClean, Thomas Smyth, Margaret Doherty

**Affiliations:** 1 Institute of Technology Sligo, Ash Lane, F91 YW50 Sligo, Ireland; 2 Cellular Health and Toxicology Research Group, Institute of Technology Sligo, Ash Lane, F91 YW50 Sligo, Ireland; 3 Northern Ireland Centre for Stratified Medicine, Biomedical Sciences Research Institute, Clinical Translational Research and Innovation Centre, Altnagelvin Area Hospital, Glenshane Road, Derry BT47 6SB, UK

**Keywords:** Alzheimer’s, biomarker, glycosylation, Aβ, tau

## Abstract

Alzheimer’s disease (AD) is of great cause for concern in our ageing population, which currently lacks diagnostic tools to permit accurate and timely diagnosis for affected individuals. The development of such tools could enable therapeutic interventions earlier in the disease course and thus potentially reducing the debilitating effects of AD. Glycosylation is a common, and important, post translational modification of proteins implicated in a host of disease states resulting in a complex array of glycans being incorporated into biomolecules. Recent investigations of glycan profiles, in a wide range of conditions, has been made possible due to technological advances in the field enabling accurate glycoanalyses. Amyloid beta (Aβ) peptides, tau protein, and other important proteins involved in AD pathogenesis, have altered glycosylation profiles. Crucially, these abnormalities present early in the disease state, are present in the peripheral blood, and help to distinguish AD from other dementias. This review describes the aberrant glycome in AD, focusing on proteins implicated in development and progression, and elucidates the potential of glycome aberrations as early stage biomarkers of AD.

## 1. Introduction

### 1.1. Alzheimer’s Disease (AD)—A Cause for Concern

There are currently over 50 million cases of dementia in the world, of which AD is the predominant form, possibly making up 60–70% of cases [1,2]. Development of AD is related to advanced age and, as life expectancy increases, it is expected that AD cases will increase four-fold by the year 2050 [3,4]. Sufferers of the disease experience deterioration of memory and cognition, which slowly worsens as the disease progresses until they are eventually unable to care for themselves [5]. From initial diagnosis, death typically occurs after 8–10 years [6].

### 1.2. AD Pathogenesis

AD is characterised by the formation of plaques composed of amyloid beta (Aβ) peptides, which are formed from abnormally processed amyloid precursor protein (APP) protein, and the presence of neurofibrillary tangles derived predominantly from hyperphosphorylated tau protein, in the brain [7]. Hyperphosphorylated tau (AD P-tau) aggregates, causing paired helical filaments (PHFs) to form, accumulation of which leads to neurofibrillary tangle formation [8]. Intraneuronal tau and extraneuronal amyloid plaques, contribute to neuronal cell death and loss of synaptic function [9,10].

Neurofibrillary tangles progressively spread from the entorhinal cortex to other parts of the brain such as the hippocampus and cerebral cortex, and these brain regions are associated with significant neuronal loss and physical shrinkage [11,12,13]. The cortex and hippocampus are strongly associated with cognition, thought, memory, learning and awareness [14,15]. Hippocampal atrophy is severe and has been used as a diagnostic and prognostic marker of AD [16,17]. Earlier diagnostic markers in these brain regions would be invaluable.

### 1.3. Treatments for AD and the Urgency of An Early Diagnosis

A deficit in the cholinergic system is a major characteristic of AD [18]. AD treatments currently available aim to correct the altered neurotransmission, but these are symptomatic as the individual will still eventually succumb to the disease [19,20]. Disease modifying treatments under investigation aim to reduce Aβ plaque formation by inhibition or modulation of secretases involved in the amyloid pathway [21,22], or increase Aβ clearance by immunological approaches [23,24]. Immunological removal of tau aggregates is also under investigation [25], in addition to prevention of tau hyperphosphorylation using kinase inhibitors, as kinases induce the hyperphosphorylation of tau [26,27]. However, the success of interventions in clinical trials has been very poor to date [28,29]. To compound matters, often current treatments are only significantly effective in mild AD sufferers, but not in moderate AD sufferers [30,31]. There is therefore a critical need to diagnose patients as early as possible so that they may derive maximum benefit from existing therapies and initiate disease modifying therapies, when they become available, as early in the disease course as possible.

### 1.4. AD Diagnosis—Invasive and Inconclusive

Aside from advanced age, the apolipoprotein E4 gene is the biggest risk factor for late onset AD; some 64% of sporadic AD sufferers and 80% of familial AD sufferers have at least one copy of the gene [32]. Mutations in APP, presenilin (PS)1 and PS2 genes almost guarantee that an individual will develop early onset AD, which occurs before 65 years of age [33]. However, early onset AD accounts for only 1–2% of AD cases [34].

At present, AD is routinely diagnosed via cognitive and learning assessments, however, this merely identifies AD as the most probable cause of symptoms which have presented. Distinguishing AD from other types of dementias is difficult as memory-related symptoms and physical changes in the brain are also observed in other neurodegenerative disorders, as well as in the normal, ageing brain [35,36,37,38]. Characteristic whole-brain and hippocampal tissue atrophy seen in AD [11] is identified using magnetic resonance imaging (MRI) and computed tomography (CT) [39], however, this atrophy is present in other forms of dementia [40,41,42]. Post mortem identification of pathological defects in the AD brain is necessary for definitive diagnosis [43].

Positron emission tomography (PET) is another imaging technique which uses radiotracers of biomarkers in an AD diagnosis [44]. 2-[18F]fluoro-2-Deoxy-D-glucose (FDG) is a common PET tracer which identifies the reduction of cerebral metabolic rates of glucose (CMRglc) seen in AD [45]. However, this disturbed CMRglc metabolism is seen in other types of dementias, making it non-specific to the disease state [46]. PET tracers of Aβ peptides, such as Pittsburg compound B, have emerged which bind specifically to amyloid plaques in the brain [47]. However, it appears that Aβ plaque formation does not correlate directly with cognitive decline [48], making it unreliable as a disease progression indicator. Promising tau-specific PET tracers have recently been developed but will require further clinical validation studies [49].

The difficulties in AD diagnosis indicates a significant clinical need to develop non-invasive diagnostic tests, ideally from peripheral blood. The popular approaches to biomarker testing today are costly neuroimaging, as discussed, and cerebrospinal fluid (CSF) analysis. CSF may be assessed for elevated total tau and AD P-tau levels, and decreased Aβ peptide levels, which requires patients to undergo a painful lumbar puncture [50]. As this review will explore, there are many reported changes in the AD glycome occurring early in the disease pathogenesis, many of which are present in the periphery and could be exploited as biomarkers of AD. Before these changes are discussed, it is important to understand glycosylation and the role it plays in human health and disease.

## 2. Glycosylation Overview

### 2.1. What is Glycosylation?

Glycosylation is an enzymatic process where glycosyltransferase enzymes use activated sugar donor molecules to attach monosaccharides to growing glycans via a glycosidic linkage [51,52,53]. Glycosidases and glycosyltransferases, involved in the synthesis of glycans, are each specific to a particular sugar and to their type of linkage [54,55]. N- and O-linked glycosylation are the most common types of protein glycosylation. O-linked glycans are linked to the hydroxyl group of threonine or serine in a protein, whereas N-linked glycans are linked to the nitrogen atom of an asparagine residue [56,57]. However there is no site specific addition of O-glycans to serine or threonine residues and the core structure of an O-glycan is variable, therefore O-glycan synthesis is not always initiated by the same glycosyltransferase [58]. The focus of this review is on N-glycans which are present in 90% of glycoproteins [59], are the most characterised of all glycans and strongly implicated in the AD pathogenesis [60,61,62,63,64]. A single enzyme known as oligosaccharyltransferase initiates glycan synthesis in the N-linked pathway. All N-glycans have a common core, and oligosaccharyltransferase specifically catalyzes the addition of the growing N-glycan to asparagine at the sequences asparagine-X-serine or asparagine-X-threonine, X being any amino acid except proline [52]. The synthesis of N-glycans begins in the endoplasmic reticulum and continues into the Golgi, occurring co- and post-translationally [52,65]. N-glycans may be either high mannose, complex or hybrid types depending on the additional sugar modifications extending from their core structure of two N-acetyl glucosamine (GlcNAc) monosaccharides and three mannoses [66,67,68,69].

### 2.2. Approaches to Glycoanalysis

Glycans may be analysed after cleavage from the respective protein, or glycoproteins may be analysed intact [70]. N-glycan cleavage may be achieved chemically by hydrazinolysis [71] or enzymatically by peptide-N4-(N-acetyl-β-glucosaminyl) asparagine amidase, typically known as PNGase F, digestion which cleaves glycans at asparagine residues, the exclusive site of N-glycosylation [72]. Since the attachment sites of glycans to the peptide backbone are different depending on the type of glycosylation, there is no single method which may be used to cleave glycans from the respective glycoproteins. Liquid chromatography (LC), porous graphitic carbon chromatography (PGC), and capillary electrophoresis are commonly used chromatographic separation methods for released glycan analysis [73].

Glycans are typically fluorescently labelled prior to LC analysis as they lack natural chromophores [74]. Hydrophilic interaction LC offers the advantage of identifying structural isomers as well as determining linkage information in some cases, which other LC modes cannot [75]. Weak anion exchange high performance liquid chromatography (HPLC) is sometimes used as an orthogonal method for the separation of similar glycans or those containing negatively charged sialic acids [76]. Databases such as GlycoStore have been established to give information related to the separated glycan structures [77].

**Figure 1:**
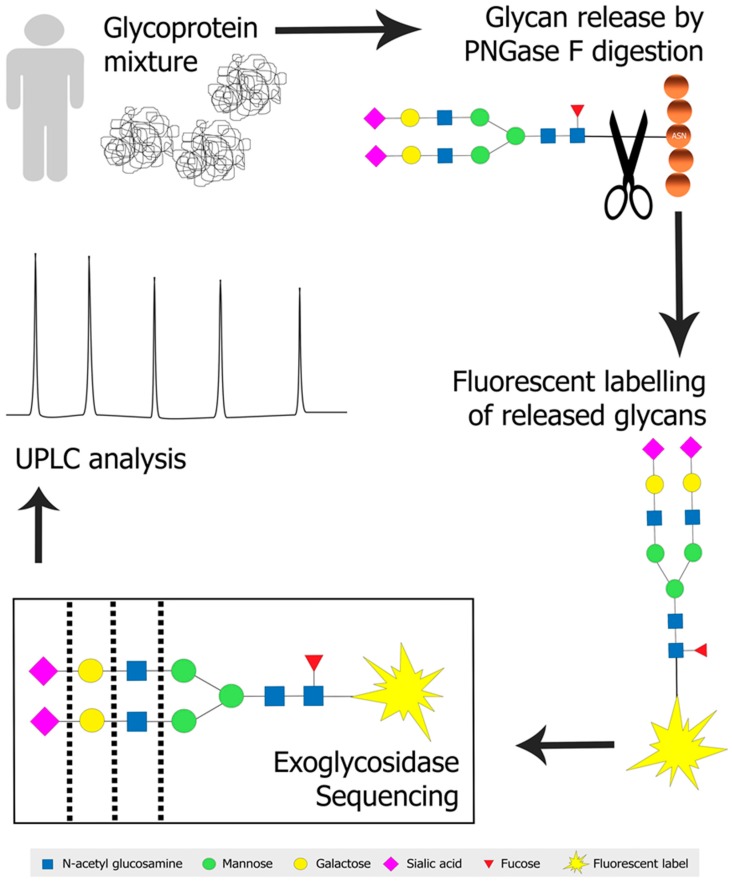
N-glycan profiling of a glycoprotein mixture. N-linked glycans are cleaved from glycoproteins by PNGase F digestion and fluorescently labelled in preparation for exoglycosidase sequencing and subsequent ultra-high performance liquid chromatography (UPLC) analysis. Terminal monosaccharides are removed from the non-reducing end of a glycan structure during an exoglycosidase digestion. Black dotted lines indicate the points of enzymatic digestion in this example of an exoglycosidase digestion. ASN, Asparagine residue in a protein chain; UPLC, ultra high performance liquid chromatography; PNGase F, peptide-N4-(N-acetyl-β-glucosaminyl) asparagine amidase. Images were created using Adobe Photoshop.

The advent of ultra-high performance liquid chromatography (UPLC) and sub-2 µm particle size columns has permitted quicker separation and improved separation capacity [78,79,80]. Separation of glycan mixtures is still difficult because isomeric glycans, that are commonly present [81], will co-elute. Exoglycosidase sequencing is therefore often used to delineate isomeric glycans identified by the initial separation, while also providing conclusive linkage information of the monosaccharides present [82,83,84]. Figure 1 illustrates a typical N-glycan profiling approach employing PNGase F digestion, fluorescent labelling, exoglycosidase sequencing and UPLC analysis of a glycoprotein mixture.

Lectins are commonly employed in glycoprofiling studies [85,86,87] and are useful in the analysis of intact glycoproteins, as well as the localisation of these glycoproteins in cells and tissues [88,89]. Mass spectrometry (MS) is another widely used and accurate method to analyse glycan structure without derivatization, which allows for analysis of intact glycoproteins [90]. MS is often used as an orthogonal technique in LC/MS or LC/MS-MS, to give an overall comprehensive analysis of glycoproteins by providing greater separation of structural or geometric glycan isomers [91,92,93,94], in addition to confirming structural linkages identified using exoglycosidase approaches [95,96]. Nuclear magnetic resonance spectroscopy is another excellent method for detailed structural analysis of glycans, but requires a relatively large quantity of purified glycans [70].

### 2.3. Glycosylation and Disease

N-glycans are vital for a multicellular organism’s survival [97,98]. Glycoproteins are on the extracellular surface of the plasma membrane, the extracellular matrix of cells, and are also secreted into bodily fluids. Almost all proteins in human serum and on mammalian cell membranes are glycosylated [59]. Glycans ensure proper protein folding, trafficking, and functionality in addition to facilitating cell signaling and cell-cell communication [99,100,101]. Changes in the physiological state may alter glycans found on glycoproteins at the same location [102,103]. Glycosylation changes also occur in many disease states. Changes in glycosylation patterns is a hallmark of cancer [104]. Breast [105], stomach [106], and liver [107] cancers all have altered serum glycoprofiles. Rheumatoid arthritis is associated with reduced IgG galactosylation and sialic acid termini [108]. Congenital disorders of glycosylation (CDGs) are a group of illnesses caused by aberrant glycosylation rooted in genetic defects in the enzymes and biomolecules involved in glycan synthesis. Over 100 CDGs exist, common examples being ALG1-CDG, PMM2-CDG and MPI-CDG, each named after the defective gene in the respective CDG [109]. Such disorders result in symptoms affecting multiple organs, commonly the brain and nervous system, due to the far reaching impact of glycosylation on the functionality of the human proteome [110,111].

## 3. Glycosylation and AD

### 3.1. Glycosylation of Proteins Implicated in AD in the Brain

Glycosylation has been implicated in AD pathology in many studies. Numerous changes have been detected in the AD brain. Immunoprecipitation and lectin blotting of β-site APP-cleaving enzyme 1 (BACE1), the N-glycosylated enzyme [112] responsible for the toxic β-secretase cleavage of APP [113], from the brains of AD patients revealed increased levels of bisecting GlcNAc, and crucially this increase was observed in both early and late stage patients [112]. A later study found that this increase in bisecting GlcNAc on BACE1 stabilises the protein under oxidative stress conditions [114], a feature characteristic of AD pathology [115].

APP is modified with O-GlcNAc [116] and studies have indicated that the mucin-type O-glycosylation of APP has a negative impact on Aβ production [117,118]. One of these studies revealed an increase in expression levels of transferases responsible for this mucin-type O-glycosylation in the early and late stage AD brain versus non-AD controls through quantitative real-time polymerase chain reaction (RT-PCR) analysis of human brain tissue [117]. N-acetylglucosaminyltransferase III (GnT-III) is the glycosyltransferase responsible for adding bisecting GlcNAc [119]. RT-PCR analysis of GnT-III mRNA revealed an increase in its expression in AD patient brains [119].

Findings suggest N-glycosylation occurs before hyperphosphorylation of tau, where non-hyperphosphorylated tau in AD brain exhibited N-glycan specific lectin staining versus an observed absence of this in normal tau in healthy controls [120]. In fact glycosylation of tau may induce hyperphosphorylation as the N-glycosylated form of the protein proved to be a better substrate for kinase than its native form [120]. The N-glycans found on phosphorylated tau and PHFs also differ, with more truncated glycans found on PHF-tau, identified in human AD brain tissue using complimentary analyses by HPLC, exoglycosidase digestions and MS [121].

Polysialylated neural cell adhesion molecule (PSA-NCAM) is recognized for its role in central nervous system development but is also reported to function in the connectivity of interneurons in the adult cerebral cortex [122]. A study revealed significantly decreased immunostaining of PSA-NCAM in the entorhinal cortex of AD brain tissue taken post mortem, relative to healthy controls. The same study found the decrease in PSA-NCAM load in the AD entorhinal cortex to be inversely proportional to hyperphosphorylated tau load [123]. This indicates a reduction in PSA-NCAM load specific to a brain region known to be severely affected in the AD pathogenesis [11,12,13], and shows that this change is potentially related in some way to increases in hyperphosphorylated tau load. Despite these findings, the identification of glycosylation changes in the AD brain is clearly not diagnostically viable.

### 3.2. Glycosylation Biomarkers of AD in the CSF and Blood

A study of the glycosylation changes in both pre-dementia and AD patient CSF using matrix-assisted laser desorption/ionization-MS showed an increase in bisect type N-glycans and a decrease in sialylated species in both pre-dementia and AD cases, indicating that AD related glycosylation changes occur before clinical symptoms manifest [124].

Detection of alterations in the AD glycome via peripheral serum and plasma would be advantageous diagnostically, given the invasive nature of CSF sampling. An obstacle in the analysis of serum is that many brain derived proteins are not present in the blood due to their inability to cross the blood brain barrier [125]. However, the N-glycoprofiles of a mouse model of neurodegeneration revealed decreases in specific glycans in the serum and cerebral cortex, and the changes correlated to learning and memory deficiencies [126]. In humans, AD patient serum exhibited increased bisect-type and highly branched glycoforms in a study which employed glycoblotting and MS analysis of patient serum samples [127]. The same study similarly showed an increase in these particular species of glycan in AD patient CSF versus non-AD controls [127]. Of note, bisect type glycans are atypical of serum, but are common “brain-type” glycans [128]. Reduced sialyltransferase activity has been observed in AD patient serum using a radio-enzymatic assay [129]. It is interesting to note that the gene for a sialic acid binding receptor, known as Siglec 33, is implicated in late-onset AD [130].

Sera levels of a bi-galactosylated core fucosylated bi-antennary glycan were shown to decrease with age in a study employing a glycoanalytical tool known as DNA sequencer-assisted, fluorophore-assisted carbohydrate electrophoresis (DSA-FACE) [131]. However, in another study employing DSA-FACE, sera levels of the same glycan were shown to decrease significantly more in AD patients in comparison to healthy controls and could additionally distinguish AD patients from other non-AD patients [132]. This is significant from a diagnostic standpoint, due to the difficulty in distinguishing AD from other neurodegenerative disorders, if it could be identified in a clinical setting using a higher throughput approach.

O-glycan changes in AD are widely reported [133,134,135,136,137,138,139,140]. Recently it was observed that neuroinflammation induced by Aβ modifies mucin-type O-glycosylation in rat hippocampus, which was observed by injection of Aβ into rat brain hippocampus followed by lectin analysis [141]. Interestingly, Aβ immunopurified from AD and non-AD CSF revealed a unique O-glycosylation of a specific tyrosine residue upon LC-MS/MS analysis, and the ratio of this tyrosine glycosylation in Aβ to the respective unglycosylated form was found to be up to 2.5 times more prevalent in the CSF of the AD patients compared to healthy and non-AD controls [142]. Aside from these general glycomic changes at play, glycosylation changes have been observed in all of the major AD related proteins, and crucially some changes are occurring at early stages in the disease state [60,61,62].

### 3.3. The Direct and Indirect Impact of BACE1 Glycosylation in AD Pathology

Cleavage of APP in neurons by α-secretase followed by γ-secretase yields a soluble peptide under normal physiological conditions [143]. Soluble APP has neuroprotective functions against excitotoxicity [144] and Aβ toxicity [145] in neurons. In AD, β-secretase and γ-secretase cleave APP sequentially, resulting in the formation of toxic Aβ peptide [146]. As stated previously, BACE1 is the enzyme responsible for the toxic β-secretase cleavage of APP [113] and is N-glycosylated [112]. Alteration of N-glycosylation sites in aspartyl protease 2, now known to be BACE1, was shown to reduce its proteolytic activity [147]. GnT-III knockout in mice resulted in reduced Aβ deposition and improved short term memory [112]. It therefore appears that aberrant N-glycosylation of BACE1 has a direct role in AD pathology. β-secretase additionally functions in protein glycosylation, specifically in cleaving sialyltransferases [148,149]. An in vitro study where a particular sialyltransferase was overexpressed in order to induce increased sialylation of APP, resulted in increased production of Aβ peptides [150]. Another in vitro study showed that overexpression of BACE1 enhances sialylation of soluble secreted glycoproteins through lectin analysis [151]. Elucidating the effects of BACE1 on the glycoprofiles of specific AD related glycoproteins may make its role more clear in the AD pathology.

### 3.4. APP Glycans as Protective Mechanisms in AD

Several studies indicate that APP sorting, secretion, transport and localisation are affected by its N-glycosylation [150,152,153,154]. Therefore glycoforms of this major AD related protein are likely to have a significant effect on the pathology of the disease. An in vitro study where mouse neuroblastoma cells were transfected with either wild-type APP or APP mutants related to AD, revealed increased levels of bisecting GlcNAc and core fucose N-glycan alterations on APP mutants compared to wild-type APP upon analysis of isolated glycans by HPLC [155]. GnT-III may act as a protective mechanism in the presence of amyloid plaques, as a study additionally revealed a decrease in Aβ production in GnT-III transfected mouse neuroblastoma cells when Aβ concentrations in culture supernatants were determined using enzyme-linked immunosorbent assay (ELISA). What is more, an increased activity of the neuroprotective α-secretase was also observed in GnT-III transfected cells [119]. A disintegrin and metalloproteinase 10 (ADAM10) is the major α-secretase in neurons responsible for normal, non-amyloidogenic cleavage of APP [156]. ADAM10 is key to neurodevelopment [157] and synaptic plasticity [158]. It interacts with APP and other proteins, and levels of ADAM10 are reduced in AD patient blood [159]. Although N-glycosylation of this glycoprotein has been shown to be crucial to its functionality by mutation of its second N-glycosylation site in vitro which caused both reduced enzymatic activity and proteolytic activation [160], its glycoprofile in AD remains elusive and warrants investigation.

### 3.5. Tau Phosphorylation is Directed by its Glycosylation

A disruption to O-GlcNAc modification of proteins in AD has been reported [134,135,136,137,138,139,140]. Tau is ordinarily O-glycosylated by single GlcNAc residues [161]. Tau protein is involved in microtubule assembly and stabilisation [162], and is hyperphosphorylated in AD [163,164]. Decreased wheat germ agglutinin (WGA) lectin blotting of tau was observed in phosphatase-inhibited human neuroblastoma cells [165]. WGA is a lectin with a high affinity for GlcNAc [166]. This indicates that hyperphosphorylation of tau, which is characteristic of AD, results in downregulation of O-linked tau glycans. As O-linked glycans attach to threonine or serine, they occupy the principal sites of phosphorylation [167]. Another study where decreased O-GlcNAcylation of tau was induced by glucose starvation of mice to mimic AD pathology resulted in increased phosphorylation of tau upon western blot analysis. This indicates that O-GlcNAcylation acts as a protective mechanism against hyperphosphorylation of tau in AD, and that the impaired glucose metabolism may be causing the reduced O-GlcNAc modification of proteins [168].

It appears that tau N-glycans are key to the stabilisation of PHF structures. The dephosphorylation and deglycosylation of tau in PHF structures increased the release of tau and restored its microtubule polymerization activity, further implicating the role of tau N-glycans in AD [169]. Tau is normally a cytosolic protein [170] and because N-glycosylation normally only occurs in secreted or membrane bound proteins [171], this may indicate a subcellular relocation of tau in AD pathogenesis.

### 3.6. Presenilin and Transmembrane Protein 59 (TMEM59) are Regulators of Protein Glycosylation

γ-secretase is partly composed of the protein presenilin [172]. Mutations in PS1 and PS2 genes are associated with early onset familial AD [33]. Acetylcholinesterase (AChE) is known to complex with presenilin, and a study conducted in transgenic mice with a PS1 mutation revealed altered mannose termini and disturbed maturation of AChE, indicating PS1 affects AChE functionality [86]. Nicastrin is the only protein in the γ-secretase complex that is N-glycosylated [173,174], and presenilin knockout cells revealed a reduction in nicastrin N-glycosylation and maturation [175]. An in vitro study where AD-related PS1 was overexpressed in a human neuroblastoma cell line identified a reduction in sialylation of neural cell adhesion molecule (NCAM) using lectin analysis [176].

TMEM59 expression is increased in late-onset AD [177]. An in vitro study showed that TMEM59 expression affects Golgi localised complex glycosylation, reducing galactosylation and sialylation of key AD related proteins such as APP, BACE1 and nicastrin using western blot analysis. The study also showed that α- and β-secretase shedding of APP and APP cell surface expression were both suppressed by TMEM59 expression [178]. These findings indicate that presenilin [86,175,176] and TMEM59 [178] regulate protein glycosylation. Further study of the effects of abnormal presenilin and upregulated TMEM59 on relevant glycoproteins may be worth investigation.

### 3.7. Apolipoprotein Glycosylation Changes in AD

Clusterin, also known as apolipoprotein J (ApoJ), is known to be associated with AD pathogenesis [179] and increased levels of clusterin are present in AD patient blood [180]. Specific glycans on clusterin were decreased in abundance in AD patients with high hippocampal atrophy relative to those with low hippocampal atrophy, analysed using LC-MS/MS analysis [181]. Apolipoprotein D (ApoD) has been shown to modulate amyloid pathology in an AD mouse model [182]. ApoD in human plasma is N-glycosylated [183]. Overall the N-glycoprofiles of the different apolipoproteins have not been investigated in detail and may be worth exploring given the indicated implications this group of proteins have in AD pathology.

### 3.8. AD-Associated Glycosylation Changes to Transferrin are Different in CSF to Serum

Increased iron concentration and disturbed iron metabolism are associated with AD [184,185]. Transferrin was identified from AD patient CSF as having reduced sialic acid termini present by lectin binding analysis and isoelectric focusing, confirmed by comparable levels of transferrin in the CSF of AD patients and controls. Combination of the reduced transferrin lectin binding with phosphorylated tau ELISA detection, this biomarker was determined to be highly specific and sensitive at distinguishing AD from other dementias [186]. Conversely, transferrin sialylation was found to be increased in AD patient serum using isoelectric focusing and immunoblotting [187]. As such, the glycoprofile of transferrin in AD appears to be altered differently in the periphery to the central nervous system.

### 3.9. Other Glycoproteins Associated with AD Pathology

Glycosylation is further implicated in other proteins associated with AD pathology. Acetylcholine has various functions in the nervous system, one of which is related to short term memory and learning [188]. A deficit in acetylcholine is associated with AD [189]. Several studies indicate glycoforms of AChE may present early in the disease state [190,191], however, conflicting findings indicate that the changes are in fact not present at early stages [192], meaning it may not be reliable early marker of AD. Reelin is a glycoprotein found in the extracellular matrix which has a role in maintaining synaptic plasticity and can reverse Aβ synaptic dysfunction [193]. It is involved in embryonic brain development [194] and adult brain functionality [195]. A change in mannose specific lectin binding to reelin has been seen in AD patient CSF in comparison to controls [196]. Neprilysin is a metalloproteinase [197] which is known to break down Aβ in the brain [198]. Neprilysin expression is downregulated in AD [199], and mutation of the N-glycosylation sites of neprilysin was shown to reduce its activity and cell surface expression [200,201]. The R47H variant of triggering receptor expressed on myeloid cells 2 (TREM2), a transmembrane protein that is reported to be associated with late-onset AD progression [202], was recently found to have an altered N-glycoprofile in comparison to the wild type. The study determined that the R47H variant has increased complex oligosaccharide alterations in comparison to the wild type, in addition to reduced stability [203].

A study in which the glycoprofiles of IgG in human blood were assessed using LC-MS/MS analysis revealed significant glycan changes in the AD group relative to controls, with a lower abundance of specific complex galactosylated and sialylated glycans observed in the AD group. Interestingly, the abundance of complex glycans in females reduced steadily from the pre-dementia cases to AD patients, whereas the opposite trend was observed in males just prior to disease onset [204]. The characteristic role of inflammation in AD [205] may explain the observed reduction in sialic acid modification of IgG seen here, as removal of sialic acids from IgG causes a shift in its function from anti-inflammation to pro-inflammation [206]. This is of interest as a readily available marker of AD which is in high abundance in the blood [207], making analyses less complex.

Regarding biomarker discovery, the focus must now turn towards a more comprehensive analysis of the AD glycome, elucidating both the well-known players in the AD pathogenesis in more detail in addition to exploring the lesser known glycoproteins implicated in the AD pathogenesis, as mentioned in this review. Crucially the methods employed should aim to provide a detailed structural analysis of glycan structures and identify their respective proteins, as opposed to the many studies of the AD glycome to date that have popularly employed approaches such as lectin analysis to gain a non-specific insight into the changes at play.

## 4. Concluding Remarks

**Figure 2:**
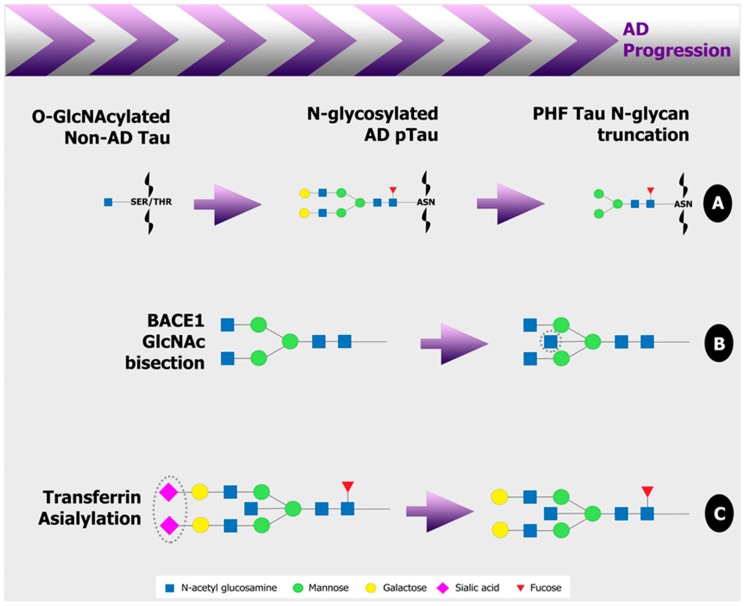
Examples of glycan alterations in disease related glycoproteins in Alzheimer’s disease (AD). (**A**) Tau, a central protein in the AD pathology, is O-GlcNAcylated which acts as a defense against hyperphosphorylation. In AD, O-glycosylation is downregulated and tau becomes uncharacteristically N-glycosylated. Both normal and hyperphosphorylated tau contain N-glycans in AD. Truncated glycans are more abundant on paired helical filaments (PHF) which are present later in the disease pathology. (**B**) Bisecting GlcNAc on β-secretase β-site APP-cleaving enzyme 1 (BACE1) occurs prior to toxic Aβ formation in AD. In contrast, GlcNAc bisection on amyloid precursor protein (APP) stimulates α-secretase production and acts as a protective mechanism against amyloid beta (Aβ) peptide formation (not shown). (**C**) Decreased terminal sialic acids are present on AD cerebrospinal fluid (CSF) glycoproteins, the major glycoprotein being transferrin which is critical to the survival of neuronal cells. Additionally, BACE1 and presenilin, a subunit of γ-secretase, both have a direct role in the sialylation of AD relevant proteins. Grey dotted lines indicate location of changes on glycan structures. Alzheimer’s disease, AD; N-acetyl glucosamine, GlcNAc; amyloid beta, Aβ; amyloid precursor protein, APP; cerebrospinal fluid, CSF; β-site APP-cleaving enzyme 1, BACE1; SER, serine; THR, threonine; ASN, asparagine; pTau, hyperphosphorylated tau; PHF, paired helical filaments. Images were created using Adobe Photoshop.

This review highlights an alteration to the glycan portion of many proteins involved in AD pathogenesis at early stages in the disease state, some examples of which are illustrated in Figure 2. With improved glycoanalytical technologies more readily available, a more detailed and comprehensive analysis of the AD glycome is the appropriate approach in future studies. Table 1 presents promising human AD biomarkers of the CSF and blood that have been reported here. Efforts of future studies should be founded in the analysis of peripheral plasma or serum samples to make glycan biomarkers of AD a viable diagnostic tool.

## Figures and Tables

**Table 1:** Candidate human biomarkers of glycosylation in AD blood and CSF.

Location	Analysis Method	Biomarker	Cohorts	Additional Comments
Serum and CSF	Glyco-blotting and MS	Increased bisect type, core fucosylated, highly branched species [127].	AD patients (*n* = 2–3) versus sex-matched non-AD controls (*n* = 2–3).	
Serum	Radio-enzymatic assay	Decreased sialyltransferase activity [129].	AD patients (*n* = 12) versus age and sex matched non-AD controls (*n* = 12).	Although both moderate and severe AD cases were assessed, there was no correlation between serum sialyltransferase activity and degree of AD. Considerable variation in the control group was observed.
Serum	DSA-FACE	Decreased bi-galactosylated core fucosylated bi-antennary glycan [132].	Population of primarily moderate/severe AD patients (*n* = 48) versus age and sex matched healthy (*n* = 149) and non-AD (*n* = 31) controls.	Desialylated serum assessed. Difference not observed between non-AD patients and age and sex matched controls. Discriminated AD patients (*n* = 48) from non-AD patients and healthy controls (*n* = 180) with a diagnostic accuracy of 85.7% ± 2.8%, 92% specificity and 70% sensitivity.
CSF	Matrix-assisted laser de-sorption/ionization-MS	Increased bisect type species and decreased sialylated species [124].	Pre-dementia (*n* = 11) and sporadic AD (*n* = 24) cases versus age matched healthy controls (*n* = 21).	40–50% of the diseased patients had this altered glycoprofile versus controls. All pre-dementia cases that converted to AD displayed an altered glycoprofile.
CSF	LC-MS/MS	Increased ratio of tyrosine linked O-glycosylated Aβ peptides to corresponding unglycosylated peptides [142].	AD patients (*n* = 6) versus non-AD patients (*n* = 7).	Patients not cognitively assessed in detail. Diagnosis based on sensitive and specific CSF biomarker detection of pathological tau and Aβ levels.
Plasma	LC-MS/MS	Decreased N-glycosylation of clusterin [181].	Mild/moderate AD patients with high hippocampal atrophy (*n* = 14) versus those with low hippocampal atrophy (*n* = 13).	N-glycans modified with mannose, galactose, sialic acid and GlcNAc. Determined that decreased glycans all present at a common N-glycosylation site on clusterin.
CSF	Lectin blotting, isoelectric focusing and MS	Decreased sialylation of transferrin [186].	Diagnosed probable AD patients (*n* = 43) versus non-AD (*n* = 13) and non-demented (*n* = 32) controls.	Combined with phosphorylated tau detection, specificity and sensitivity was 88.4% and 92.3%, respectively. CSF transferrin levels did not differ between groups.
Serum	Isoelectric focusing and immuno-blotting	Increased penta- and hexa-sialylation of transferrin [187].	AD patients (*n* = 11) versus non-demented, age-matched controls (*n* = 14).	
CSF	Lectin blotting	Increased mannosylated glycans on reelin [196].	AD patients (*n* = 11) versus non-demented, age- and sex-matched controls (*n* = 9).	Combining two lectin stains increased discrimination of AD from controls. 10 of 11 AD cases were below an arbitrary cutoff point, and 7 of 9 controls were above this cutoff.
Plasma	LC-MS/MS	Decreased complex, galactosylated and sialylated glycans on IgG [204].	AD patients (*n* = 31) versus non-demented controls (*n* = 26).	One such bi-antennary, complex, bi-galactosylated glycan decreased in females (*n* = 93) steadily prior to disease onset from earlier to later stage cases, but an inverse trend was true for males (*n* = 65).

Mass spectrometry, MS; Alzheimer’s disease, AD; DNA sequencer-assisted, fluorophore-assisted carbohydrate electrophoresis, DSA-FACE; cerebrospinal fluid, CSF; liquid chromatography, LC; amyloid beta, Aβ; N-acetyl glucosamine, GlcNAc.
